# Karyotype’s Rearrangement in Some Hybrids of the Orchidinae Subtribe

**DOI:** 10.3390/plants13202838

**Published:** 2024-10-10

**Authors:** Alessio Turco, Robert Philipp Wagensommer, Antonella Albano, Pietro Medagli, Saverio D’Emerico

**Affiliations:** 1 Faculty of Education, Free University of Bozen-Bolzano, 39042 Brixen-Bressanone, Italy; alessio.turco@unibz.it; 2 Department of Biological and Environmental Sciences and Technologies, University of the Salento, 73100 Lecce, Italy; antonella.albano@unisalento.it (A.A.); pietro.medagli@unisalento.it (P.M.); 3 “Aldo Moro” University of Bari, 70125 Bari, Italy; sdeme@yahoo.it

**Keywords:** *Anacamptis*, FISH, hybridization, karyomorphology, marker chromosomes, morphometric parameters, *Ophrys*, *Serapias*

## Abstract

Based on our karyological findings in the *Anacamptis* Rich., *Ophrys* L., and *Serapias* L. genera, we have identified chromosomal markers within some hybrids and elucidated their interrelationships. Mitotic chromosomes of fifteen taxa were analyzed using the conventional Feulgen staining method. Only for *Anacamptis ×gennarii* (Rchb. f.) H.Kretzschmar, Eccarius & Dietr. [*A. morio* (L.) R.M.Bateman, Pridgeon & M.W.Chase × *A. papilionacea* (L.) R.M.Bateman, Pridgeon & M.W.Chase] and its parental species were some data obtained and reported with the banding method with Giemsa, Hoechst 33258 fluorochrome, and the FISH techniques. Our research involved new chromosomal measurements of fifteen taxa, including six hybrids, along with schematic representations. Morphometric parameters, i.e., M_CA_ and CV_CL_, were used to evaluate karyotype asymmetry. Of meaning were the analyses performed on chromosomal complements of selected hybrids, which distinctly revealed marker chromosomes present in one or both putative parental species. Among the parents identified in some hybrids, *Ophrys tenthredinifera* Willd. has shown some interest due to the presence in its karyotype of a pair of chromosomes (n.1) showing a notable secondary constriction on the long arm. Indeed, one of the homologs is clearly distinguishable in the analyzed hybrids, where it clearly emerges as one of the putative parents. Given the challenges in detecting certain karyomorphological features within the Orchidinae subtribe using alternative methods, such as Giemsa C-banding or fluorescence banding, the Feulgen method remains valuable for cytogenetic characterization. It helps us to understand the genomes of hybrids and parental species, thus contributing to a deeper understanding of their genetic composition.

## 1. Introduction

Hybridization is an important process in the evolution of plants [1,2,3]. New hybrid lineages must establish reproductive isolation and a unique ecological niche to overcome genetic mixing and competition from parental species [4]. Hybridization can result in new species of the same ploidy level (homoploid hybrid speciation) or different ploidy levels (allopolyploid hybrid speciation) [5,6,7]. Otherwise, homoploid hybrid speciation is rarer than the allopolyploid mode, as hybrids are generally sterile [8]. However, if they colonize new habitats, particularly those that are not congenial to the parental species, hybrids are likely to show better fitness and successfully establish themselves in new ecological niches [9]. Thus, homoploid hybrids are largely reported in novel habitats that are not occupied by parental species [2,10,11].

In higher plants, two aspects of interspecific hybridization barriers are observed, including prezygotic and postzygotic barriers, which may occur during or after syngamy [12,13,14]. Hybridization can restructure the genome and then modify phenotypic traits that affect ecological interactions, i.e., this is an important factor for interactions with pollinators [15,16,17]. However imprinting and epigenetic regulation in interspecific hybrid failure have been suggested in addition to ploidy differences [18].

The Orchidaceae family shows, within the Angiosperms, the greatest specific richness with approximately 26,000 species, showing also a great number of interspecific and intergeneric hybrids. These hybrids generally demonstrate a “transitional” morphology from their parental species [19,20]. In entomophilous plants, the high specific diversity is justified by a series of reproductive strategies aimed at attracting pollinators, the majority of which are not species specific, such as in the “food deceptive strategies” [21].

In the genus *Ophrys* L., a group characterized by about 369 taxa including varieties [22], it has been widely demonstrated that plants mimic, through visual, tactile, and olfactory signals, the female partner of a specific pollinator [23,24,25,26]. In this context, some studies have also widely demonstrated that olfactory signals are the key stimulus in this insect–orchid interaction, especially in the attraction at great distances through the emission of “Biological Active Compounds”, substances like the pheromones produced by the pollinator females [25,26,27], promoting the phenomenon of pseudocopulation. Vereecken et al. [28] have shown how the copulative activity of the insect is inconsistent, which always starts in the expected position (cephalic or abdominal) but constantly changes position, favoring the uptake of pollinodes in different positions and favoring hybridization even between taxa of different groups. In this way, the hybrid derived produces “new” compounds which can produce errors in the hybridization processes and, in some cases, favor speciation [29,30,31,32].

Cytological research, including karyotype analysis, can be considered an important and useful approach used for evaluating taxonomic relationships and phylogenetic classification. Indeed, karyological data from the studies in recent years represent essential information on ecological characteristics, taxonomy, evolution, and phylogeny of the Orchidinae group [33,34,35,36].

Even though Orchidinae have a large representative and wide distribution area, only 23 genera have been cytogenetically studied so far [37,38]. The chromosome numbers in the members of the subtribe Orchidinae range from 2n = 32 to 2n = 42, and some wild species are polyploid [34,39,40].

The current karyological knowledge on spontaneous Orchidaceae allows us to express some considerations on the affinities between the basic karyotypes of most of the genera of the subtribe Orchidinae. Among the genera examined, there are well-distinct basic karyotypes in *Anacamptis* Rich., *Ophrys*, and *Serapias* L., which are characterized by marker chromosomes. Indeed, the karyotype of the analyzed species shows that, despite the similarity between taxa, there are differences in the morphology of chromosomes. In addition, differences in the amount and distribution of heterochromatin have been found [38,39,41]. Although the mentioned genera have been subjected to numerous cytogenetic analyses and karyotypes have been described for different populations, the chromosome complement of numerous interspecific or intergeneric hybrids seems to be comparatively less studied [34]. A cytogenetic characterization of hybrids and their parental species would aid in a better understanding of their species status and, as previously mentioned, focus on the importance of hybridization in speciation processes. On the other hand, in the literature, there are few works of a molecular nature in which hybridization and its consequences were analyzed compared to the numerous natural hybrids described in recent years.

Based on our previous works, we considered some peculiar natural hybrids, growing wild in Apulia, and their supposed parents that are characterized by the presence of some marker chromosomes observed in cytotaxonomic investigations.

Therefore, the aim of the present study was to highlight the chromosomal markers in some hybrids and their reciprocal parentals to compare with the earlier results.

In addition, the karyotypes of the examined taxa have been analyzed using several parameters such as variations in length, arm ratio, and centromeric asymmetry indices [42,43].

Considering the limited cytogenetic information, the increase in chromosomal data may provide valuable phylogenetic signals about Orchidinae diversity. In this study, the mitotic chromosomes of some hybrids were analyzed using conventional Feulgen staining methods, and only for *Anacamptis ×gennarii* (Rchb.f.) H.Kretzschmar, Eccarius & Dietr. and its parental species we present some data with banding with Giemsa, Hoechst 33258 fluorochrome, and FISH, with the aim of providing new data that will improve the knowledge on Orchidinae cytogenetics.

## 2. Results

**Figure 1:**
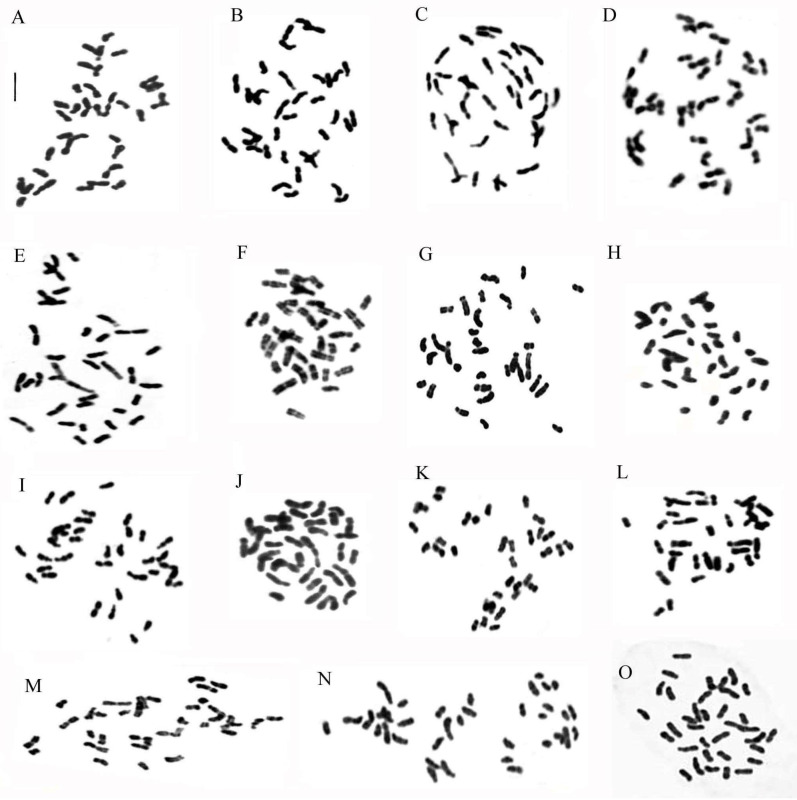
Metaphase chromosomes of (**A**) *Ophrys apulica*, 2n = 36; (**B**) *O. tenthredinifera*, 2n = 36; (**C**) *O. incubacea*, 2n = 36; (**D**) *O. bombyliflora*, 2n = 36; (**E**) *Anacamptis morio*, 2n = 36; (**F**) *A. collina*, 2n = 36; (**G**) *A. papilionacea*, 2n = 32; (**H**) *Serapias parviflora*, 2n = 36; (**I**) *Ophrys ×salentina*, 2n = 36; (**J**) *Ophrys ×franciniae*, 2n = 36; (**K**) *Ophrys ×sommieri*, 2n = 36; (**L**) *Anacamptis ×gennarii*, 2n = 34; (**M**) *Anacamptis ×semisaccata* nothosubsp. *murgiana*, 2n = 36; (**N**) *×Serapicamptis nelsoniana*, 2n = 36; (**O**) *Ophrys tardans*, 2n = 36. Scale bar = 5 µm.

Table 1 lists all 15 analyzed taxa, highlighting the karyotype and related parameters, while the metaphase chromosomes of all the analyzed taxa are shown in Figure 1.

### 2.1. Karyomorphological Analysis

#### 2.1.1. *Ophrys* ×*salentina O.Danesch* & *E.Danesch*

**Figure 2:**
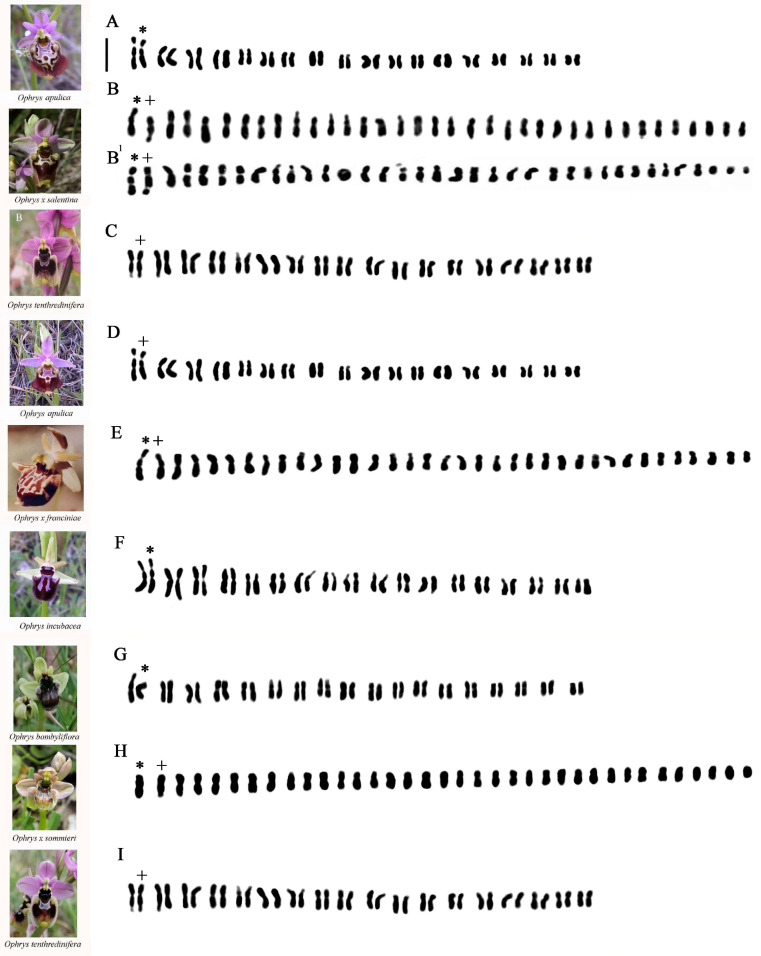
Karyotypes of (**A**) *Ophrys apulica*; (**B**,**B^1^**) *O. ×salentina* (two karyotypes of different specimens); (**C**) *O. tenthredinifera*; (**D**) *O. apulica*; (**E**) *O. ×franciniae*; (**F**) *O. incubacea*; (**G**) *O. bombyliflora*; (**H**) *O. ×sommieri*; (**I**) *O. tenthredinifera*. Asterisks and plus signs indicate marker chromosomes observed in the analyzed taxa. Scale bar = 5 µm.

In this interspecific hybrid, individual chromosome lengths ranged from 1.05 to 1.25 in the shortest and from 4.23 to 4.35 in the longest chromosomes. In the hybrid karyotype (Figure 2B,B^1^), it is possible to observe a homologous relative to the first pair of *O. apulica* (O.Danesch & E.Danesch) O.Danesch & E.Danesch (Figure 2A, *), consisting of a chromosome with a secondary constriction on the short arm and one of the homologs of the first couple present in *O. tenthredinifera* Willd., consisting of a chromosome with an evident constriction on the long arm characteristic of this latter species (Figure 2C, +). In addition, three chromosomes with a secondary constriction on the short arm can be noted, which are always observed in *O. tenthredinifera* (Figure 2C).

#### 2.1.2. *Ophrys* ×*franciniae Bianco*, *Medagli*, *D’Emerico* & *Ruggiero*

Individual chromosome lengths ranged from 1.72 to 1.95 in the shortest and from 4.07 to 5.20 in the longest chromosomes. In the hybrid karyotype (Figure 2E), a chromosome with a secondary constriction on the short arm like the first pair observed in the diploid karyotype of *O. apulica* can be observed in the second position (Figure 2D, +). Always in the hybrid karyotype, in the first position, we observe a chromosome with a large linear satellite present in the chromosomal set of the parental *O. incubacea* Bianca (Figure 2F, *).

#### 2.1.3. *Ophrys* ×*sommieri E.G.Camus ex Cortesi*

Individual chromosome lengths ranged from 1.68 to 1.96 in the shortest and from 3.35 to 3.47 in the longest chromosomes. In the hybrid (Figure 2H), it is possible to observe one of the homologs of the first couple present in *O. bombyliflora* Link (Figure 2G, *) and one of the homologs of the first couple present in *O. tenthredinifera*, consisting of a chromosome with a constriction on the long arm (Figure 2I, +).

#### 2.1.4. *Anacamptis* ×*semisaccata nothosubsp. murgiana* (*Medagli*, *D’Emerico*, *Ruggiero* & *Bianco*) *H.Kretzschmar*, *Eccarius* & *H.Dietr.*

**Figure 3:**
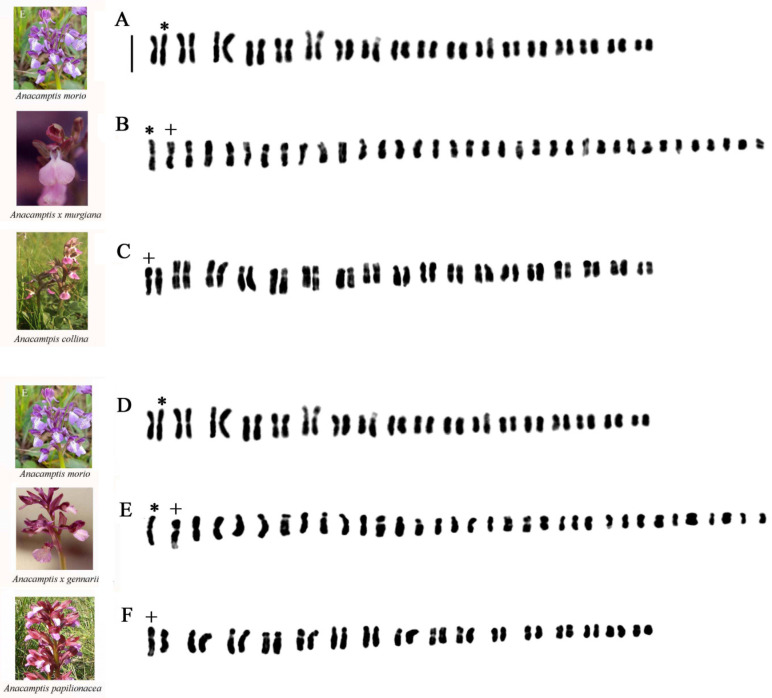
Karyotypes of (**A**) *Anacamptis morio*; (**B**) *A. ×semisaccata nothosubsp. murgiana*; (**C**) *A. collina*; (**D**) *A. morio*; (**E**) *A.* ×*gennarii*; (**F**) *A. papilionacea*. Asterisks and plus signs indicate marker chromosomes observed in the analyzed taxa. Scale bar = 5 µm.

In this interspecific hybrid individual chromosome, lengths ranged from 1.76 to 1.80 in the shortest and from 3.98 to 4.74 in the longest chromosomes. In the hybrid karyotype (Figure 3B), we observed three metacentric chromosomes and two chromosomes with a secondary constriction on the short arm present in the chromosomal set of the parental *Anacamptis morio* (L.) R.M.Bateman, Pridgeon & M.W.Chase (Figure 3A). In Figure 3B +, it is possible to observe in the second position one of the homologs of the first couple present in *A. collina* (Banks & Sol. ex Russell) R.M.Bateman, Pridgeon & M.W.Chase (Figure 3C, +), consisting of a submetacentric chromosome with a secondary constriction on the long arm, which is characteristic of the parental species.

#### 2.1.5. *Anacamptis ×gennarii*

**Figure 4:**
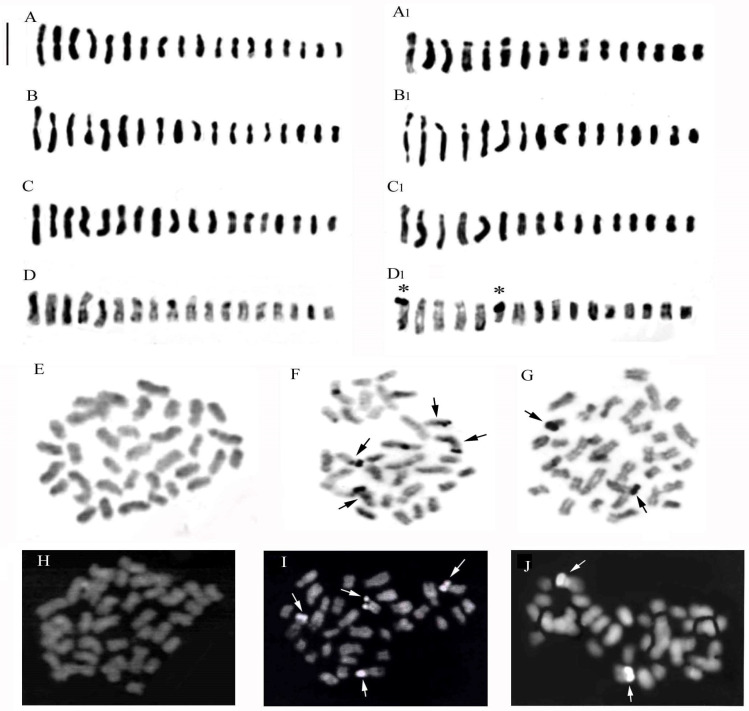
Somatic metaphases of *Anacamptis* ×*gennarii* with possible separation of the haploid complement of *A. morio* (**A**–**D**) and haploid complement of *A. papilionacea* (**A1**–**D1**). It is possible to notice in the two kits a notable variation of the chromosomes following notable rearrangements during meiosis. (**D**,**D1**) Somatic metaphases of *Anacamptis* ×*gennarii* staining with Giemsa C-band (asterisks indicate telomeric bands). (**E**–**G**) Staining with Giemsa C-band (arrows indicate telomeric bands): *A. morio* (**E**), *A. papilionacea* (**F**), *A. ×gennarii* (**G**). (**H**–**J**) Somatic metaphases of *A. morio* (**H**), *A. papilionacea* (**I**), *A.* ×*gennarii* (**J**) treated with the fluorochrome Hoechst 33258. In *A. papilionacea,* we can observe four chromosomes with telomeric bands; in *A.* ×*gennarii*, we observe only two chromosomes with telomeric bands belonging to *A. papilionacea* (arrows). Differently, *A. morio* does not show any important banding. Scale bar = 5 µm.

**Figure 5:**
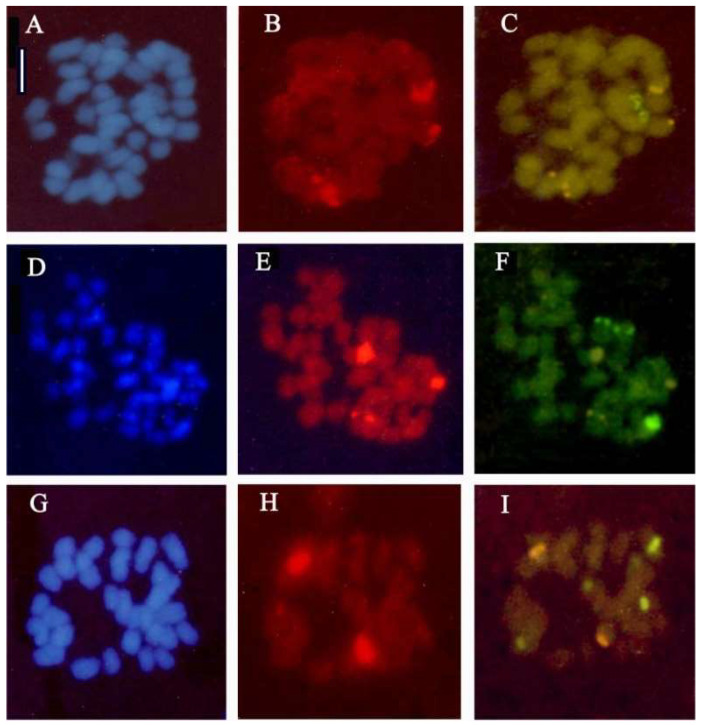
In situ hybridization applied to the chromosomes of *Anacamptis morio*, *A.* ×*gennarii*, and *A. papilionacea*. Blue DAPI staining shows chromosomal DNA, respectively, in *A. morio* (**A**), *A.* ×*gennarii* (**D**), and *A. papilionacea* (**G**). Red and green signals show sites of hybridization of 18S-25S rDNA and 5S rDNA: in *A. morio* (**C**), four 18S-25S rDNA sites and two 5S rDNA sites; in *A.* ×*gennarii* (**F**), three 18S-25S rDNA sites and three 5S rDNA sites; in *A. papilionacea* (**I**), two 18S-25S rDNA sites and four 5S rDNA sites. Red signals show sites of hybridization of 18S-25S rDNA (**B**,**E**,**H**) in the three taxa. Scale bar = 5 µm.

*Anacamptis ×gennarii* is a very widespread and frequent interspecific hybrid in Apulia, and it is found in populations where the parental species, i.e. *A. morio*, with chromosomal number 2n = 36, and *A. papilionacea* (L.) R.M.Bateman, Pridgeon & M.W.Chase, with 2n = 32, abound in sympatria, allowing to highlight a chromosomal number 2n = 34 intermediate between the parental ones. Individual chromosome lengths ranged from 1.54 to 1.63 in the shortest and from 4.28 to 4.44 in the longest chromosomes. In the hybrid karyotype (Figure 3E), we observe numerous metacentric chromosomes and two chromosomes with a secondary constriction on the short arm present in the chromosomal set of the parental *A. morio* (Figure 3D). Moreover, in the second position, there is a secondary constriction, and one per arm is present in *A. papilionacea*. In addition, there are numerous submetacentric and subtelocentric chromosomes that are characteristic of the parental species *A. papilionacea* (Figure 3E, +). For this hybrid, we examined numerous specimens karyologically, and based on the parental karyotypes, we tried to separate the possible chromosomes of the two chromosomal complements (Figure 4). The difference between the karyotypes of the two parents allowed for a separation of the haploid complements (Figure 4A,A1,B,B1,C,C1,D,D1). In this hybrid, in addition to the Feulgen method, we also used the banding technique with Giemsa, Hoechst 33258 fluorochrome, and FISH (Figure 4D,D1,E–J and Figure 5). The first two techniques made it possible to highlight in the hybrid some marker chromosomes present in the parents.

### 2.1.6. ×*Serapicamptis nelsoniana* (*Bianco*, *D’Emerico*, *Medagli* & *Ruggiero*) *J.M.H.Shaw*

**Figure 6:**
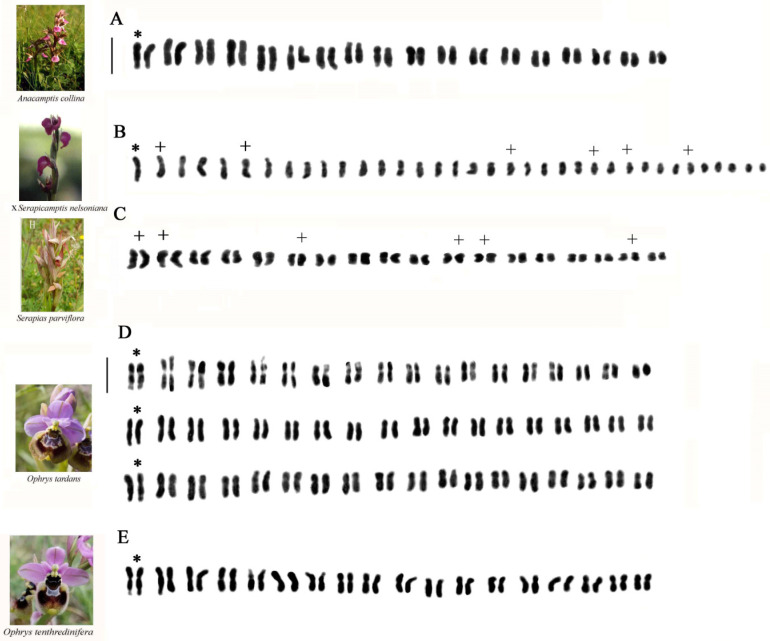
Karyotypes of (**A**) *Anacamptis collina*; (**B**) ×*Serapicamptis nelsoniana*; (**C**) *Serapias parviflora*; (**D**) *Ophrys tardans* (Three karyotypes in different specimens); (**E**) *Ophrys tenthredinifera*. Asterisks and plus signs indicate marker chromosomes observed in the analyzed taxa. Scale bar = 5 µm.

In this intergeneric hybrid, individual chromosome lengths ranged from 1.50 to 1.67 in the shortest and from 3.59 to 5.03 in the longest chromosomes. In the hybrid karyotype (Figure 6B), we observe three metacentric chromosomes and one of the homologs of the first couple consisting of a submetacentric chromosome with a secondary constriction on the long arm present in the chromosomal set of the parental *Anacamptis collina* (Figure 6A, *). Numerous small chromosomes are characteristic of the parental *Serapias parviflora* Parl. (Figure 6C, +). It is interesting to observe the different dimensions of the chromosomes between the two parents. Indeed, *Anacamptis collina* are larger than those present in the complement of *S. parviflora*.

#### 2.1.7. *Ophrys tardans O.Danesch* & *E.Danesch*

In this species, the chromosome lengths ranged from 1.50 to 5.03 in the longest chromosomes. The karyotype morphology consists of 34 m + 2 sm chromosomes (Figure 6D). Pair 1 possesses a secondary constriction on the long arm identical to that of *Ophrys tenthredinifera* (Figure 6E).

### 2.2. Fluorescence In Situ Hybridization (FISH) in Anacamptis ×gennarii and Its Parental Species

The method of detecting recurring DNA sequences was applied to the hybrid *A. ×gennarii* and its parental species, *A. morio* and *A. papilionacea*. In situ hybridization allowed the localization of three 18S-25S rDNA signals and three 5S rDNA sites, which confirms the parents of the hybrid *A. ×gennarii* being *Anacamptis morio* and *A. papilionacea* (Figure 5).

### 2.3. Diagram of the Morphometric Parameters

**Figure 7:**
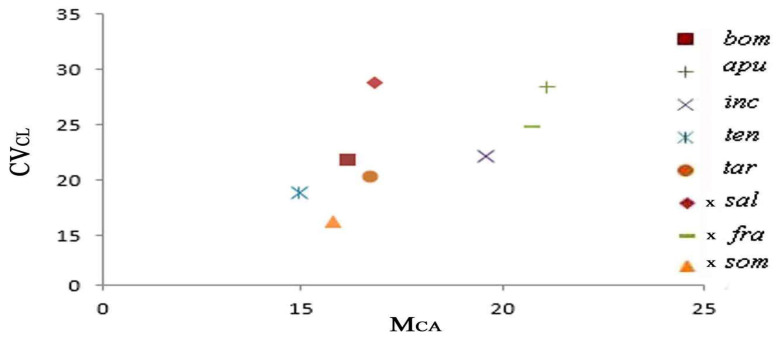
Diagram of the M_CA_ and CV_CL_ values of the karyotypes of the taxa examined in *Ophrys* species and hybrids. In the diagram, it is interesting to note the parameter values of the hybrids intermediate to the supposed parents. Codes: see Table 1.

**Figure 8:**
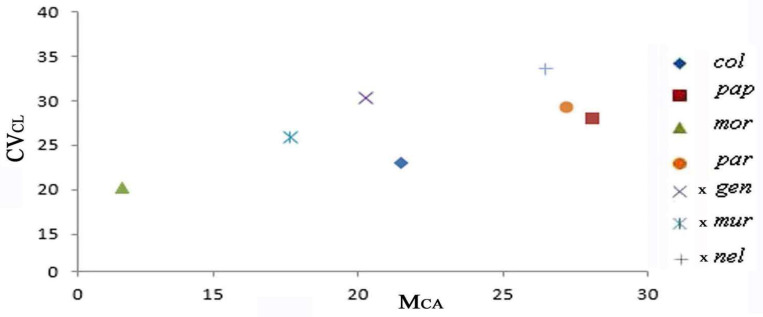
Diagram of the M_CA_ and CV_CL_ values of the karyotypes of the taxa examined in *Anacamptis*, *Serapias* species, and hybrids. In the diagram, it is interesting to note the parameter values of the hybrids intermediate to the supposed parents. Codes: see Table 1.

We used the asymmetry indices M_CA_ and CV_CL_ to produce the diagrams in Figure 7 and Figure 8, which highlight selected species, and each are represented by a distinct color. The diagrams perfectly show the intermediate karyological parameters observed in the hybrids and present in the parental karyotypes.

## 3. Discussion and Conclusions

### 3.1. Interspecific Hybrids in Ophrys

Previous cytological investigations in *Ophrys* have indicated x = 18 as the basic haploid chromosome number [44,45,46]. Bianco et al. [46,47] provided the first karyotypes for *Ophrys,* indicating that the “basic” karyotype of *Ophrys* showed three chromosome pairs with evident secondary constriction, where a long satellite on the short arm characterized the first pair. However, a variation in the size of the long satellite in pair 1 has been observed in the *Ophrys* species analyzed. Moreover, the taxa within the genus have karyotypes that can be arranged in series, showing a progressive orientation from the symmetrical to the moderately asymmetrical type [39].

As mentioned above, numerous species of the genus *Ophrys* show a first pair characterized by an evident secondary constriction, which varies in the sections into which the genus is divided. For example, a comparison of karyotype structures showed that in the *O. fusca*–*O. lutea*–*O. omegaifera* complex, the first pair is characterized by the presence of a medium secondary constriction on the short arm [48]. In *Araniferae*, *Fuciflorae*, and *Apiferae* sections, the first pair of chromosomes differs markedly in satellite size where the satellite present in the short arm is much larger than in the other sections [39,49,50,51]. Moreover, based on chromosome morphology, in the *Tenthrediniferae* section, the presence of a secondary constriction on the long arm of the first chromosome pair has been noted [41,50]. The group formed by *O. bombyliflora*, *O. tenthredinifera*, and *O. tardans* (*O. bombyliflora*–*O. tenthredinifera* complex) seems to behave as a transitional group between the *O. fuciflora*–*O. tetraloniae*–*O. oestrifera* complex and the *Araniferae* and *Fuciflorae* sections [48,52,53,54].

The application of the C-banding technique to the species of *Ophrys* examined revealed that all of them possess small centromeric bands, with some taxa characterized by chromosomes with telomeric and subtelomeric bands. It is interesting to notice that using this technique, it is possible to observe that the species of this genus belonging to different sections showed clear differences in the amount of total heterochromatin [49]. However, through the data obtained with Giemsa C-banding, it was not possible to identify the taxa of hybrid origin.

Based on the data currently in our possession relating to the first pair of chromosomes, it is possible to identify three standard karyotypes in the genus *Ophrys* with a first pair of chromosomes that display a different secondary constriction. In fact, this first pair clearly distinguishes the three groups mentioned and can be used to discriminate some chromosomes in the karyotype of the hybrids with traditional techniques.

The hybrid *Ophrys ×salentina* is not very widespread, and this seems to be due to the limited range of *O. apulica* compared to that of *O. tenthredinifera* and, therefore, to their small overlapping of the ranges. In this hybrid, it is quite easy to identify the homolog of the first pair present in *O. tenthredinifera*, consisting of a chromosome that is currently observed only in this species in the *Ophrys* group, with an evident secondary constriction on the long arm in the first pair.

Also, in *Ophrys ×sommieri,* the identification of the marker chromosomes was facilitated by showing a homolog present in the parental *O. tenthredinifera*.

On the other hand, *Ophrys ×franciniae* shows a remarkable polymorphism due to the great variability of *O. apulica* as well as introgressive phenomena in progress. In this hybrid, it is possible to identify the different karyomorphologies of a homolog present in the parental species *O. incubacea* and *O. apulica*. In fact, in *Ophrys incubacea*, the first pair is characterized by a chromosome with an evident secondary constriction on the short arm, which is different from the first pair of *O. apulica*, showing a first pair characteristic of the *Ophrys holosericea* group.

The three hybrids showed values of morphometric parameters perfectly intermediate between the parental species.

### 3.2. Interspecific Hybrids in Anacamptis

Previous records in the *Anacamptis* genus indicate mainly diploids with 2n = 2x = 36 and polyploid cytotypes with 2n = 3x = 54 and 2n = 4x = 72 chromosomes. Species with uniformly symmetrical karyotypes, comprising mainly metacentric chromosomes, such as *A. morio*, *A. longicornu* (Poir.) R.M.Bateman, Pridgeon, and M.W.Chase, *A. laxiflora* (Lam.) R.M.Bateman, Pridgeon and M.W.Chase, and *A. pyramidalis* (L.) Rich., showed little constitutive heterochromatin [39,55]. *A. collina* and *A. palustris* (Jacq.) R.M.Bateman, Pridgeon, and M.W.Chase possessed karyomorphological characteristics, which separate them from the species of the *O. morio* cluster. A. *collina* shows a large number of chromosome pairs bearing secondary constrictions. Differently, *O. papilionacea*, the only species with chromosome number 2n = 32, has quite an asymmetrical karyotype. The somatic chromosome complement includes a characteristic pair of chromosomes (n. 1) with two secondary constrictions, one per arm [39,55].

In the Orchidinae subtribe, cytogenetic studies on natural hybrids are very rare, and only in two interspecific hybrids of the *Anacamptis* s.l. were useful results obtained for a more detailed understanding of the chromosomal complements of hybrids [56]. In this work, based on the most in-depth knowledge of chromosomal complements obtained in numerous species of the genus, it was possible, on a karyological basis, through some traditional methods, to separate the chromosomes of the parental species in hybrids.

In the hybrid *Anacamptis ×gennarii*, the karyotype of the hybrid was extremely variable in the numerous specimens examined, thus confirming the relevant differences between the complements of the supposed parentals, which are largely responsible for the sterility of the hybrid specimens and which play a relevant role in the absence of the processes of introgression. Interestingly, this process has been confirmed by molecular studies using nuclear ITS1 and AFLP (amplified fragment length polymorphism) [57]. The hybridization process in the *A. morio × A. papilionacea* hybrid was of relevance; in fact, worthy of note was to observe in the populations examined specimens showing some characteristics of a single relative or intermediate characteristics among the parental species in addition to other individuals that presented entirely new characteristics. In this interesting hybrid, both traditional cytogenetic methods and in situ fluorescence were used [39,58]. However, in this work, we have shown interesting results in highlighting some marker chromosomes with traditional techniques.

Another interesting case of interspecific hybridization identified through karyological analyses was observed in Cassano Murge (Bari) in some hybrid specimens, originating from the cross between *Anacamptis collina* (2n = 36) and *A. morio* (2n = 36). Out of four hybrid specimens identified, with morphological characteristics intermediate between the supposed parental ones, three showed a diploid chromosomal number of 2n = 36 while the fourth showed a triploid number of 2n = 3x = 54. The karyotype of this last hybrid specimen, of allopolyploid origin, showed 18 pairs of chromosomes typical of *A. collina*, while the haploid kit belongs to *A. morio* [56]. The complement of this hybrid showed many chromosomes with secondary constriction on the short arm and long arm, which were observed also in the karyotypes of parental species *A. collina* and *A. morio*.

The two hybrids also showed values of morphometric parameters perfectly intermediate between the parental species.

### 3.3. Intergeneric Hybrid between Anacamptis and Serapias

The observations on morphological and karyological bases in the intergeneric ×*Serapicamptis nelsoniana* have led to the identification of the parents in *Anacamptis collina* and *Serapias parviflora*. It is interesting to note the rarity of the hybrid despite the two parental species having very large and largely overlapping ranges, which is probably due to the autogamy processes of *S. parviflora* and the early flowering of *A. collina*. The karyological analyses showed chromosomal number 2n = 36 like the number found in the parental species. In the first moment, the karyotype was erroneously constructed by composing the chromosomes in pairs. Currently, since numerous karyological research was carried out in the genus *Anacamptis* and the genus *Serapias*, it was possible to identify, with a good probability, some characteristics and marker chromosomes present in the parental species. In fact, the species of the genus *Anacamptis* have a quite distinct karyomorphology from the species of the genus *Serapias* [39].

### 3.4. Homoploid Hybridization or Epigenetic Origin?

Finally, *Ophrys tardans*, originally described as a hybrid between *O. tenthredinifera* and *O. candica* (E.Nelson ex Soó) H.Baumann & Künkele, forms populations in general that are completely distinct from the parental species. Therefore, some questions arise regarding *Ophrys tardans*. The origin of this taxon is given by homoploid hybridization, or is it a possible morphospecies of *O. tenthredinifera* derived from epigenetic factors? These karyological findings appear to support the idea that the origin of *Ophrys tardans* is probably derived from epigenetic factors. Indeed, in the karyotype of *O. tardans,* the first pair is perfectly like *O. tenthredinifera*. Furthermore, in the diagram, the Mca and CVcl parameters are very close to *O. tenthredinifera.* The karyological findings obtained in this study combined with the molecular and genomic FISH study will contribute significantly to answering the questions about the origin of this interesting taxon.

## 4. Materials and Methods

### 4.1. Natural Hybrids and Parental Species

The present study examined wild taxa growing in Apulia (Italy). The studied specimens were photographed, without uprooting them, but some immature ovaries were taken from the flowers. The growing sites of the natural hybrids, of which some are rare to find, and their parental species, are reported in Table 2. The hybrids were identified in the field due to their morphological characteristics, and their hybrid nature was then confirmed by the karyological analyses carried out. Except for the intergeneric hybrids, during the samplings, only plants with morphological intermediate features were sampled (i.e., the presence or length of the gibbae, design of the macula, color and shape of the labellum).

Morphological analyses were also made using the original description of the hybrids. Parental species were taken into pure populations, i.e., without visible signs of introgression with other species. The hybrids analyzed in this paper are *Ophrys ×salentina* (*Ophrys apulica* × *O. tenthredinifera*), *Ophrys ×franciniae* (*O. apulica* × *O. incubacea*), *Ophrys ×sommieri* (*O. bombyliflora* × *O. tenthredinifera*), *Anacamptis ×semisaccata* nothosubsp. *murgiana* (*A. collina × A. morio*), *Anacamptis ×gennarii* (*A. morio × A. papilionacea*), and ×*Serapicamptis nelsoniana* (*Anacamptis collina × Serapias parviflora*).

*Ophrys ×salentina* is a natural hybrid derived from the crossing between *O. apulica* and *O. tenthredinifera* established by Danesch O. and Danesch E. following a discovery made in Apulia in 1970. The hybrid is not very widespread, and this seems to be due to the limited range of *O. apulica* compared to that of *O. tenthredinifera* and, therefore, to their small overlapping, both of the ranges and of the flowering periods.

*Ophrys ×franciniae* is a hybrid derived from the cross between *O. apulica* and *O. incubacea* and was first found in 1988 near Cassano Murge (Bari, Apulia). The hybrids show a remarkable polymorphism due to the great variability of *O. apulica* as well as introgressive phenomena in progress. In this case, too, the difference in the flowering period between the two parental species made this hybrid not very widespread.

*Ophrys ×sommieri* is a natural hybrid derived from the cross between *O. bombyliflora* and *O. tenthredinifera*. This hybrid is more abundant and easier to recognize.

*Anacamptis ×semisaccata* nothosubsp. *murgiana* is an interesting and very rare case of interspecific hybridization identified through karyological analyses observed in Cassano Murge (Bari, Apulia) in some hybrid specimens, originating from the cross between *Anacamptis collina* and *A. morio*. The orchidological component at the discovery site was essentially composed of the parental species *A. collina* and *A. morio*.

*Anacamptis* ×*gennarii* is a very widespread and frequent interspecific hybrid in Apulia in populations where the parental species *Anacamptis morio* and *A. papilionacea* are abound in sympatria. For the analyses of this hybrid, we chose meadows where only the parental species *A. morio* and *A. papilionacea* were abundant, as well as numerous specimens of *A.* ×*gennarii*.

×*Serapicamptis nelsoniana* is a very rare intergeneric hybrid originating from the crossing between a species of the genus *Anacamptis* and a species of the genus *Serapias* (*Anacamptis collina* × *Serapias parviflora*) and was found for the first time in 1985 near Nardò (Lecce, Apulia). The orchidological component at the site of the discovery of the intergeneric hybrid was composed of *Anacamptis morio*, *A. papilionacea*, *Neotinea lactea* (Poir.) R.M.Bateman, Pridgeon & M.W.Chase, *Serapias lingua* L., *S. parviflora*, and *S. vomeracea* (Burm.f.) Briq. *Ophrys tardans*, originally described as a hybrid between *O. tenthredinifera* and *O. candica*, was later recognized as a species endemic to Salento (a small portion of Apulian territory), as confirmed in the recent checklist of the Italian native vascular flora [59]. It is very rare and localized, forming populations in general completely distinct from the putative parental species.

### 4.2. Cytological Analysis

The karyological analyses of the hybrids are based on new data obtained through the analyses carried out and presented in this paper and revised and updated data based on further results obtained on numerous species of the genera *Ophrys*, and *Anacamptis* s.l. and *Serapias* are also presented.

#### 4.2.1. Feulgen Technique, Giemsa C-Banding, and Hoechst Fluorochrome

Methods for cytological analysis used in this paper include the Feulgen stain for chromosomal counting and karyomorphological analysis, Giemsa C-band staining to detect constitutive heterochromatin, Hoechst 33258 fluorochrome staining to identify A-T-rich regions, and FISH (Figure 1, Figure 2, Figure 3, Figure 4, Figure 5 and Figure 6). The material used for cytological analysis consisted of immature ovaries. The study of chromosomes through immature ovaries is advantageous both for the protection of orchids and for the presence of somatic meristematic cells and EMC for meiotic division.

Mitotic chromosomes were observed in tissues of immature ovaries. These were pre-treated with 0.3% colchicine at room temperature for 2 h. For Feulgen staining, they were fixed in 3:1 (*v*/*v*) ethanol–glacial acetic acid and stored in the deep freezer for up to several months. Hydrolysis was made at 20 ± 2 °C in 5.5 N HCl for 20 min [60]. The material was then stained in freshly prepared Feulgen stain. At least five well-spread chromosome plates were selected for karyotype analysis.

For C-banding, immature ovaries were fixed in 3:1 (*v*/*v*) ethanol–glacial acetic acid and stored in the deep freezer for up to several months. Subsequently, they were squashed in 45% acetic acid; coverslips were removed using the dry-ice method, and the preparations were air dried overnight. Slides were then immersed in 0.2 N HCl at 60 °C for 3 min, thoroughly rinsed in distilled water, and then treated with 4% Ba(OH)_2_ at 20 °C for 4 min. After thorough rinsing, they were incubated in 2× SSC at 60 °C for 1 h. They were then stained using 3–4% Giemsa (BDH) at pH 7.

For Hoechst 33258 staining, squash preparations were made up as they were for C-banding and were then stained in a 2 µg/mL dye solution in a pH 7 McIlvaine buffer for 5 min, rinsed, and mounted in 1:1 *v*/*v* buffer–glycerol [61].

#### 4.2.2. Fluorescence In Situ Hybridization

Among the analyzed hybrids, only *Anacamptis* ×*gennarii* is relatively widespread, coexisting with its parental species. Therefore, the fluorescence in situ hybridization (FISH) analyses were conducted only for the natural hybrid *A.* ×*gennarii* and parental species (*A. morio* and *A. papilionacea*) in relation to the widespread presence of specimens of these three taxa. In addition, it was easier to analyze meristematic tissues compared to other groups belonging to the Orchidinae subtribe.

Five well-spread metaphase plates were examined with the FISH technique. For fluorescence in situ hybridization, the ribosomal sequences 18S-5.8S-25S (pTa71—red signals) and 5S (pTa794—green signals) were used as probes. Clone pTa71 was labeled with rhodamine-4-dUTP by nick translation, while pTa794 was labeled with digoxigenin11-dUTP using a polymerase chain reaction. The former contains a 9kb EcoBl repeat unit of 18S-5.8S-25S rDNA and intergenic spacer regions isolated from *Triticum aestivum* L. [62], and the latter corresponds to a complete 410 bp 5S gene unit, containing the 5S gene and intergenic spacer regions and isolated from *Triticum aestivum* [63]. The pre-treatment of slides and the FISH procedure followed the protocol in Heslop–Harrison [64]. The chromosomes and DNA probes were denatured together at 70 °C for 5 min, and hybridization was performed at 37 °C overnight. After hybridization, the coverslips were removed in 2× SSC at room temperature and then washed thoroughly for 10 min in 20% (*v*/*v*) formamide in 0.1× SSC at 42 °C to remove sequences with less than 85% homology; the slides were then incubated in immunofluorescent reagents. For detection of the digoxigenin-labeled probe, the slides were equilibrated in 4× SSC/0.1% (*v*/*v*) Tween 20 and blocked in 5% (*w*/*v*) bovine serum albumin in 4× SSC/0.1% (*v*/*v*) Tween 20 for 5 min. The slides were incubated with sheep anti-digoxigenin antibody conjugated with FITC in a moist chamber at 37 °C for 1 h. The slides were washed in 4× SSC/Tween 20 for 3 × 5 min and subsequently counterstained with DAPI prior to observation. They were finally mounted in an antifade solution AF1 (Citifluor) and examined with a Leitz epifluorescence microscope with single and triple band-pass filters. The resulting images were processed with free image-editing software, applying the functions to the whole image.

### 4.3. Chromosome Numbers and Karyotype Parameters

Due to the rarity of some natural hybrids, karyomorphometric analyses on the studied specimens have been limited, in some cases to a single specimen (e.g., ×*Serapicamptis nelsoniana*), unlike the numerous specimens of the parental species.

Chromosome measurements were performed using the freeware IdeoKar 1.2 (http://agri.uok.ac.ir/ideokar/index.html, accessed on 10 May 2023). Chromosome pairs were identified and arranged based on length. The nomenclature used for describing karyotype composition followed Levan et al. [65]. Karyotype morphometric characteristics were evaluated by calculating the haploid complement, while the karyotype asymmetry indices M_CA_ (Mean Centromeric Asymmetry) and CV_CL_ (Coefficient of Variation of Chromosome Length) were used for the evaluation of karyotype asymmetry. Moreover, CV_CI_ (Coefficient of Variation of the Centromeric Index) was used to evaluate heterogeneity in the position of the centromeres [42,66,67].

Diagrams of the Mca/CVcl values of the karyotypes were generated through the OpenOffice 4.1.14 program.

### 4.4. Nomenclature

The nomenclature used for the Orchidaceae family follows, depending on the taxa considered, both GIROS [68] and Delforge [22].

## Figures and Tables

**Table 1:** Taxon, code, chromosome number, formula, and morphometric parameters (average values). THL = total chromosome length of the haploid complement; MCA = Mean Centromeric Asymmetry; CVCL = Coefficient of Variation of Chromosome Length; CVCI = Coefficient of Variation of Centromeric Index. Chromosome abbreviations: *m*, metacentric; *sm*, submetacentric; *st*, subtelocentric.

Taxon	Code	Chromosome Number	Formula	THL	M_CA_	CV_CL_	CV_CI_
*Ophrys bombyliflora* Link	bom	36	32 *m* + 4 *sm*	43.61	16.20	21.72	10.13
*O. apulica* (O.Danesch & E.Danesch) O.Danesch & E.Danesch	apu	36	20 *m* + 16 *sm*	46.86	21.08	28.46	15.67
*O. incubacea* Bianca	inc	36	26 *m* + 10 *sm*	42.91	19.55	22.24	12.42
*O. tenthredinifera* Willd.	ten	36	30 *m* + 6 *sm*	50.96	14.95	18.94	10.58
*O. tardans* O.Danesch & E.Danesch	tar	36	32 *m* + 4 *sm*	41.64	16.72	20.26	10.17
*Ophrys* ×*salentina* O.Danesch & E.Danesch	sal	36	26 *m* + 10 *sm*	45.71	16.84	28.87	14.70
*Ophrys* ×*franciniae* Bianco, Medagli, D’Emerico & Ruggiero	fra	36	22 *m* + 14 *sm*	51.51	20.78	24.76	19.03
*Ophrys* ×*sommieri* E.G.Camus ex Cortesi	som	36	32 *m* + 4 *sm*	44.80	15.81	16.29	13.82
*Anacamptis collina* (Banks & Sol. ex Russell) R.M.Bateman, Pridgeon & M.W.Chase	col	36	18 *m* + 18 *sm*	48.47	21.43	22.85	19.86
*A. papilionacea* (L.) R.M.Bateman, Pridgeon & M.W.Chase	pap	32	16 *m* + 12 *sm* + 4 *st*	40.70	28.11	27.77	25.68
*A. morio* (L.) R.M.Bateman, Pridgeon & M.W.Chase	mor	36	30 *m* + 6 *sm*	44.94	11.86	20.12	10.61
*Anacamptis ×gennarii* (Rchb.f.) H.Kretzschmar, Eccarius & Dietr.	gen	34	22 *m* + 10 *sm* + 2 *st*	44.52	20.21	30.24	21.60
*Anacamptis* ×*semisaccata* nothosubsp. *murgiana* (Medagli, D’Emerico, Ruggiero & Bianco) H.Kretzschmar, Eccarius & H.Dietr.	sem	36	26 *m* + 8 *sm* + 2 *st*	54.58	17.61	25.91	21.27
*Serapias parviflora* Parl.	par	36	16 *m* + 18 *sm* + 2 *st*	40.87	27.21	29.12	20.07
×*Serapicamptis nelsoniana* (Bianco, D’Emerico, Medagli & Ruggiero) J.M.H.Shaw	nel	36	16 *m* + 14 *sm* + 6 *st*	48.05	26.44	33.49	26.29

**Table 2:** Taxon, code, number of specimens, and growing sites (collecting sites of the ovaries) of the investigated samples.

Taxon	Code	Number of Specimens	Growing Sites (Collecting Sites of the Ovaries)
*O. bombyliflora* Link	Bom	5	S. Cataldo, LE; Cassano Murge, BA; Santeramo in Colle, BA (Apulia, Italy)
*O. apulica* (O.Danesch & E.Danesch) O.Danesch & E.Danesch	Apu	5	S. Cataldo, LE; Cassano Murge, BA (Apulia, Italy)
*O. incubacea* Bianca	Inc	4	Cassano Murge, BA (Apulia, Italy)
*O. tenthredinifera* Willd.	Ten	8	S. Cataldo, LE; Le Cesine, LE; Porto Selvaggio, LE; Cassano Murge, BA (Apulia, Italy)
*O. tardans* O.Danesch & E.Danesch	Tar	6	S. Cataldo, LE; Le Cesine, LE; Otranto, LE (Apulia, Italy)
*Ophrys ×salentina* O.Danesch & E.Danesch	Sal	4	S. Cataldo, LE; Le Cesine, LE; Porto Selvaggio, LE; Cassano Murge, BA (Apulia, Italy)
*Ophrys ×franciniae* Bianco, Medagli, D’Emerico & Ruggiero	Fra	2	Cassano Murge, BA (Apulia, Italy)
*Ophrys ×sommieri* E.G.Camus ex Cortesi	Som	3	Bosco Rauccio, LE; Cassano Murge, BA; Santeramo in Colle, BA (Apulia, Italy)
*Anacamptis collina* (Banks & Sol. ex Russell) R.M.Bateman, Pridgeon & M.W.Chase	Col	4	Nardò, LE; Cassano Murge, BA (Apulia, Italy)
*A. papilionacea* (L.) R.M.Bateman, Pridgeon & M.W.Chase	Pap	6	Adelfia, BA; Cassano Murge, BA; Conversano, BA (Apulia, Italy)
*A. morio* (L.) R.M.Bateman, Pridgeon & M.W.Chase	Mor	5	Adelfia, BA; Cassano Murge, BA; Conversano, BA (Apulia, Italy)
*Anacamptis ×gennarii* (Rchb.f.) H.Kretzschmar, Eccarius & Dietr.	Gen	5	Adelfia, BA; Cassano Murge, BA; Conversano, BA (Apulia, Italy)
*Anacamptis ×semisaccata* nothosubsp. *murgiana* (Medagli, D’Emerico, Ruggiero & Bianco) H.Kretzschmar, Eccarius & H.Dietr.	Sem	2	Cassano Murge, BA (Apulia, Italy)
*Serapias parviflora* Parl.	Par	4	Nardò, LE; Cassano Murge, BA (Apulia, Italy)
×*Serapicamptis nelsoniana* (Bianco, D’Emerico, Medagli & Ruggiero) J.M.H.Shaw	Nel	1	Nardò, LE (Apulia, Italy)

## Data Availability

Data are contained in the article.
